# Non-Epileptic Convulsions in Growing Children

**Published:** 1903-02-14

**Authors:** 


### NON-EPILEPTIC CONVULSIONS IN GROWING CHILDREN.

Lightly as people often regard convulsions in infants, the occurrence of apparently similar convulsive attacks in children who have left infancy behind them and have attained school age is very apt to be regarded as a manifestation of epilepsy and to be labelled as such, much to the detriment of the child. Dr. Eustace Smith,[1] however, points out that convulsive attacks, similar to those that are so often seen in rickety or highly neurotic infants as a result of teething or other form of reflex irritation, may be found in children, even as late as the eleventh or twelfth year, being in them also due to pure reflex worry. The children thus affected are usually members of families which show distinctly neurotic tendencies, and are themselves highly strung, excitable, and easily moved to tears although by no means necessarily spiritless or timid. They may have suffered from convulsions during infancy, but often their special nervous susceptibility is of later origin, and seems to be quite independent of the rickety state with which convulsions during infancy are so often associated. Hence on the occurrence of convulsions in a growing child, it is most important before regarding such a case as one of epilepsy, with all its gloomy prospects, to consider carefully all the circumstances of the attack with a view to discover some exciting cause to which the seizures may be traced. It will often be found that there have been premonitory symptoms common to all the attacks, the patient having been for some hours or days before the seizure very obviously out of health. The seat of the trouble is usually in the alimentary canal, being often due to undigested or fermenting food. As a rule the attacks are separated by long intervals, during which the health, although perhaps not perfectly satisfactory, is fair. Sometimes the attack consists of a single seizure which is not repeated in the same bout of illness, while in other cases an attack may comprise many seizures, the whole group lasting several consecutive days, or even being spread over a period of weeks, then ceasing for a time to return again in the same way after a longer or shorter interval. The special feature of these attacks is, that they invariably coincide either with a digestive disturbance or with some other form of local irritant, and cease when this local condition is removed. Among the conditions which may conspire to produce these convulsive attacks may be mentioned adenoids, scybalous masses in the intestines, and eye-strain set up by hypermetropia or astigmatism. The great thing is to remember that these attacks are more allied to infantile convulsions than to epilepsy. If a child, whether he be or be not of known neurotic tendencies, begins to suffer from periodical fits, we must not too hastily assume that the case is a serious one, or that the patient is or is likely to become epileptic. If we are able to connect the seizure with a local irritant or passing derangement of organs we have reason to hope, and even to anticipate, that the removal of the disturbing influence may restore quiet to the nervous system. Instead then of flying at once to bromides and other nervine sedatives we should search without delay for the exciting cause and adopt prompt measures for its removal. Doubtless cases which ultimately develop the characteristics of true epilepsy do occasionally begin in the same way, that is in attacks occurring only when the health is deranged; but in the large majority of instances, where the fit occurs in a child who is the subject of a temporary digestive upset, we may reasonably expect that by judicious treatment the seizures may be put a stop to and the health be permanently restored. Many cases, Dr. Eustace Smith declares, warrant him in saying that young persons who have been subject to such seizures, even up to as late as 12 years old, may grow up into perfectly healthy adults without showing any sign of having suffered from a weakness which has long become a thing of the past. The importance of such a statement from so well known an authority is manifest.

[1] Lancet, Jan. 24.

